# En Masse Resection of Pancreas, Spleen, Celiac Axis, Stomach, Kidney, Adrenal, and Colon for Invasive Pancreatic Corpus and Tail Tumor

**DOI:** 10.1155/2013/376035

**Published:** 2013-09-15

**Authors:** Koray Kutluturk, Abdul Hamid Alam, Cuneyt Kayaalp, Emrah Otan, Cemalettin Aydin

**Affiliations:** ^1^Inonu University, Malatya, Turkey; ^2^Rabia Balkhi Hospital, Kabul, Afganistan; ^3^Department of Surgery and Liver Transplantation Institute, Inonu University, Malatya, Turkey

## Abstract

Providing a more comfortable life and a longer survival for pancreatic corpus/tail tumors without metastasis depends on the complete resection. Recently, distal pancreatectomy with celiac axis resection was reported as a feasible and favorable method in selected pancreatic corpus/tail tumors which had invaded the celiac axis. Additional organ resections to the celiac axis were rarely required, and when necessary it was included only a single extra organ resection such as adrenal or intestine. Here, we described a distal pancreatic tumor invading most of the neighboring organs—stomach, celiac axis, left renal vein, left adrenal gland, and splenic flexure were treated by en bloc resection of all these organs. The patient was a 60-year-old man without any severe medical comorbidities. Postoperative course of the patient was uneventful, and he was discharged on postoperative day eight without any complication. Histopathology and stage of the tumor were adenocarcinoma and T4 N1 M0, respectively. Preoperative back pain of the patient was completely relieved in the postoperative period. As a result, celiac axis resection for pancreatic cancer is an extensive surgery, and a combined en masse resection of the invaded neighboring organs is a more extensive surgery than the celiac axis resection alone. This more extensive surgery is safe and feasible for selected patients with pancreatic cancer.

## 1. Introduction

Unlike pancreatic head tumors, distal pancreas tumors rarely cause jaundice. Mostly these lesions cause upper abdominal pain radiating to the low back as a result of local invasion to the celiac axis. Left-sided pancreatic tumors are usually delayed in diagnosis because of the difficulties at the differential diagnosis of low back pain which is very frequent in elderly people. Therefore, liver metastasis, peritoneal carcinomatosis, or major vascular invasion to celiac axis or superior mesenteric artery is frequent at the time of diagnosis and up to 75% of the tumors are evaluated as unresectable [1–6]. Providing a more comfortable life and a longer survival for pancreatic corpus/tail tumors without metastasis depends on the complete resection of those tumors [5–7].

Our aim was to present a case of a distal pancreatic tumor invading most of the neighboring organs—stomach, celiac axis, left renal vein, left adrenal gland, and *transvers mesocolon*—which was treated by en bloc resection of all these organs. Recently, distal pancreatectomy with celiac axis resection was reported as a feasible and favorable method in selected pancreatic corpus/tail tumors [1, 4, 6, 7]. As far as we know, however, there were very rare cases that required resection of so many organs combined with celiac axis as en masse for pancreatic corpus/tail tumors.

## 2. Case Presentation

**Figure 1:**
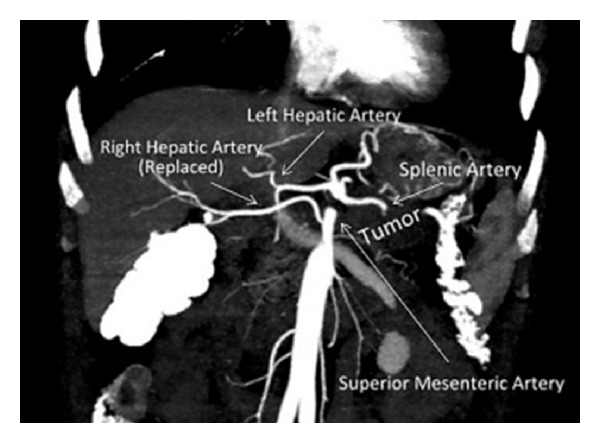
Pancreatic tumor and the main abdominal vascularities.

**Figure 2:**
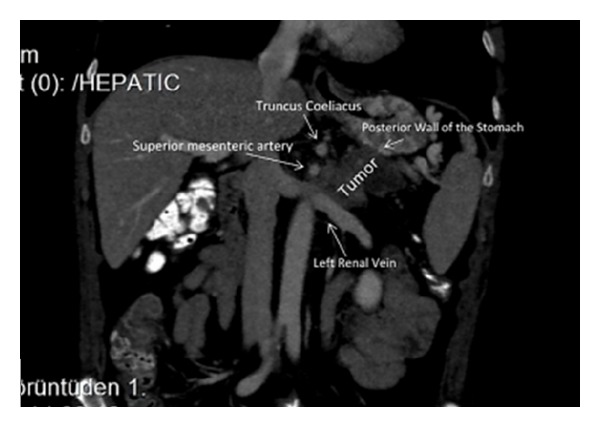
Invasion of the posterior gastric wall and the left renal vein.

**Figure 3:**
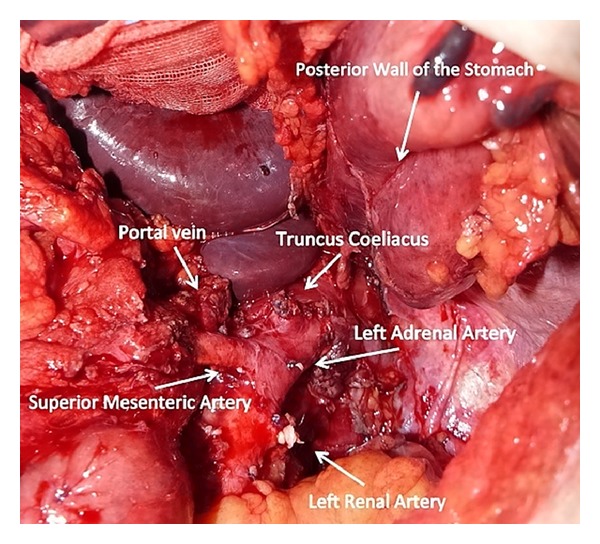
Intraoperative view after en masse resection of the tumor with neighbouring organs.

A 60-year-old male patient was admitted with complaints of low back pain and loss of appetite. He had a history of several diagnostic and therapeutic interventions for low back pain. At last, an abdominal computed tomography revealed a solid lesion (5 × 3.5 cm) at the pancreatic corpus and tail, which had indistinguishable fatty interplains with superior mesenteric artery (Figure 1). Right hepatic artery was observed to be originating from proximal portion of the superior mesenteric artery (Figure 2). The patient and his relatives were informed that optimum clarification of the tumor whether it was resectable or not could be done only during surgical exploration due to the close relationship of the tumor and the main abdominal branches of the aorta. The patient was operated with his relatives' consent. During exploration it was noted that the mass had invaded transvers mesocolon on the splenic flexure site and at the level of Treitz ligament. The mass was in close contact with superior mesenteric vein as well. Following division of the gastrocolic ligament, it was noted that the mass was adherent to the celiac axis and invaded the left renal vein, but it was completely released from the superior mesenteric artery. In this state (situation), distal pancreatectomy (pancreatic tissue on the left portion of portal vein), splenectomy, celiac axis resection, partial gastrectomy, left nephrectomy, left adrenalectomy, and left hemicolectomy with end colostomy by closure of the distal stump were performed (Figure 3). Before deciding on celiac axis resection, we occluded the celiac axis with a vascular clamp and checked and felt the hepatic arterial pulsation at the hepatic hilum. Tumor adhesions to the celiac axis were not divided and en-bloc resection of the celiac axis was done (along) with the tumor. For gastric wall invasion, at first, we only performed a localized posterior gastric wall resection, but this procedure was completed to near-total gastrectomy due to ischemia of the remaining part of stomach. Gastric continuity was obtained by a Roux-en-Y gastrojejunostomy. Duration of the whole surgical procedure was 470 minutes.

**Figure 4:**
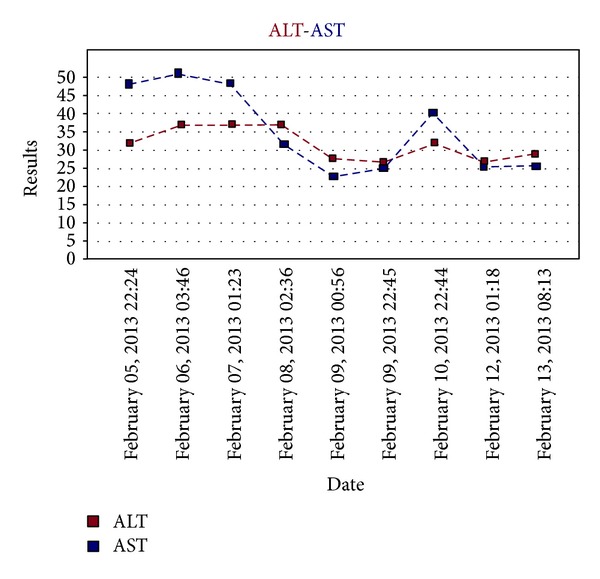
Postoperative transaminase levels of the patient (operated on February 5, 2013).

*On postoperative day one*, the patient was extubated, and the nasogastric tube was removed. During the whole postoperative course liver enzyme levels were always within normal range (Figure 4). Pancreatic leakage was not observed. On postoperative day eight, the patient was discharged uneventfully and free of any complication.

Histopathological examination reported as pancreatic ductal adenocarcinoma with an extensively invaded peri-pancreatic tissue with perineural, lymphatic, and venous invasion. It had invaded adrenal gland, stomach wall, mesocolon, and renal vein. There was no histopathologically confirmed celiac axis and splenic tumor invasions, but there were fibrotic adhesions to the celiac axis. Of 29 lymph nodes resected, two had tumor metastasis; among nine lymph nodes resected around superior mesenteric artery, none of them had metastasis. Following medical oncology consultation, the patient was given chemotherapy, and after that he was planed for reversal of end colostomy. His back pain was completely relieved in the postoperative period. While this paper was prepared, the patient was at the end of the third month of his followup. 

## 3. Discussion

One of the aims of preoperative radiological evaluation of an invasive intra-abdominal cancer is to identify patients with unresectable or incurable advanced disease in order to prevent unnecessary laparotomy. Our previous studies have demonstrated that preoperative radiological evaluation sometimes may be inadequate for determining tumour invasion of the main vasculature and for detecting peritoneal metastasis. Patients who are considered fit to undergo tumour resection should not be denied operative assessment on the basis of radiological findings of adjacent organ involvement, except in cases of large circular mass invasion. Since there is a high rate of false positives and false negatives for tumoural invasion on radiological evaluation, there may be a risk of undertreating patients who are actually operable [8, 9]. Preoperative radiological evaluation of this case could not distinguish the margin between the pancreatic mass and the left lateral border of the superior mesenteric artery. However, there was not any preoperative finding about the celiac axis invasion which was detected during operation, possibly because of enlarged celiac lymph nodes and their close relationships between the celiac axis and the tumor. Posterior gastric wall was invaded. Liver and the other intra-abdominal organs were free of metastasis. Although the tumor is suitable for being accepted as unresectable in this situation, studies on locally advanced nonmetastatic pancreatic corpus and tail tumors demonstrate that complete resection of the tumor with surrounding tissues provides a longer survival and better patient comfort [5–7]. Surgical treatment was offered to our patient as the tumor was not metastatic.

During surgery, we could dissect out the tumor from the superior mesenteric artery without leaving any affected tissue around the artery. However, celiac resection was performed due to the adherence of the tumor there. Celiac resection was first described by Graham et al. which was performed on a patient who underwent gastrectomy for gastric tumor [10]. Later, Nimura et al. described the en-bloc resection of celiac axis with splenectomy for pancreatic corpus tumors [11]. Possible risks of celiac resection with distal pancreatectomy are acute liver failure due to hepatic ischemia, ischemic gastropathy due to gastric ischemia, gastric necrosis, haemorrhage, and perforation [4, 12]. Celiac resection removes directly gastric and hepatic arterial perfusion, and following this procedure, these organs are perfused via pancreaticoduoneal collaterals between superior mesenteric artery and gastroduonenal artery [2, 13, 14]. Therefore, preoperative assessment of these arteries is necessary before the surgical procedure. When required, preoperative celiac axis embolisation with digital subtraction angiography (DSA) can be performed in order to increase hepatic perfusion [7, 14, 15]. In our case, there was not any visible apparent collateral between gastroduodenal artery and the superior mesenteric artery in the reconstructed computed tomography scans. However, preoperative embolisation was not performed as the patient had aright hepatic artery originating from the proximal portion of the superior mesenteric artery. At surgery, hepatic arterial blood flow was checked by palpation of the liver hilum as well. Postoperative followup was uneventful without any sign of hepatic ischemia, and liver function test results were within normal levels.

In our case, the tumor had invaded the gastric wall posteriorly. Initially, gastric wedge resection was performed, however, almost complete ischemia following celiac axis resection led to high-subtotal gastrectomy. Various studies reported similar to our case that gastric ischemia may develop following celiac axis resection and some stated that left gastric artery might be reanastomosed to celiac stump for prevention of gastric ischemia [1]. In our case, we preferred high subtotal gastrectomy due to the risks of reanastomosis of the left gastric artery in an ischemic and partially resected stomach. 

During the postoperative followup, the patient described that his back pain was reduced markedly. Celiac axis resection was reported to provide such a pain relief [16, 17]. We did not use any additional surgical method for pain control such as alcohol injection around the celiac plexus. As a result, en-bloc resection of coeliac axis combined with resection of several neighboring organs for nonmetastatic, locally advanced pancreatic corpus and tail tumor invading celiac axis is possible to perform safely. It is too early for us to say anything about the benefit of this surgery on our patient survival; however, we can clearly say that such aggravated extensive surgery was technically feasible, and postoperative pain relief of the patient was remarkable.
